# Multivariate techniques enable a biochemical classification of children with autism spectrum disorder versus typically‐developing peers: A comparison and validation study

**DOI:** 10.1002/btm2.10095

**Published:** 2018-06-19

**Authors:** Daniel P. Howsmon, Troy Vargason, Robert A. Rubin, Leanna Delhey, Marie Tippett, Shannon Rose, Sirish C. Bennuri, John C. Slattery, Stepan Melnyk, S. Jill James, Richard E. Frye, Juergen Hahn

**Affiliations:** ^1^ Dept. of Chemical & Biological Engineering Rensselaer Polytechnic Institute Troy NY 12180; ^2^ Center for Biotechnology and Interdisciplinary Studies, Rensselaer Polytechnic Institute Troy NY 12180; ^3^ Dept. of Biomedical Engineering Rensselaer Polytechnic Institute Troy NY 12180; ^4^ Dept. of Mathematics Whittier College Whittier CA 90602; ^5^ Arkansas Children's Research Institute Little Rock AR 72202; ^6^ Dept. of Pediatrics University of Arkansas for Medical Sciences Little Rock AR 72205; ^7^ Barrow Neurological Institute at Phoenix Children's Hospital, Phoenix, AZ 85013 and University of Arizona College of Medicine Phoenix AZ 85004

**Keywords:** autism spectrum disorder, biomarkers, multivariate statistical analysis

## Abstract

Autism spectrum disorder (ASD) is a developmental disorder which is currently only diagnosed through behavioral testing. Impaired folate‐dependent one carbon metabolism (FOCM) and transsulfuration (TS) pathways have been implicated in ASD, and recently a study involving multivariate analysis based upon Fisher Discriminant Analysis returned very promising results for predicting an ASD diagnosis. This article takes another step toward the goal of developing a biochemical diagnostic for ASD by comparing five classification algorithms on existing data of FOCM/TS metabolites, and also validating the classification results with new data from an ASD cohort. The comparison results indicate a high sensitivity and specificity for the original data set and up to a 88% correct classification of the ASD cohort at an expected 5% misclassification rate for typically‐developing controls. These results form the foundation for the development of a biochemical test for ASD which promises to aid diagnosis of ASD and provide biochemical understanding of the disease, applicable to at least a subset of the ASD population.

## 1 INTRODUCTION

Autism Spectrum Disorder (ASD) encompasses a large group of early‐onset developmental disorders that are collectively characterized by deficits in social interaction and communication as well as the expression of restricted, repetitive behaviors and interests.^1^ ASD is currently estimated to affect 1 in 68 children,^2^ incurring an annual expenditure of $268 billion in the United States,^3^ and this prevalence continues to rise.^2^, ^4^ The median age of diagnosis is still 50 months in the United States,^2^ with some evidence that this age is lowering.^5^ In the United Kingdom, the average age of diagnosis is estimated to be 55 months with no evidence of decreasing.^6^ Part of this discrepancy can be attributed to the difference in positions on national screening between the two countries: in 2007, the American Academy of Pediatrics in the U.S. called for general screening followed by a comprehensive evaluation for ASD by 24 months^7^, ^8^ while the National Health Service in the U.K. advocates against universal screening.^9^ Camarata^10^ indicates that this difference in recommendations largely lies in the reliability of ASD diagnosis at 24 months^11^, ^12^ and that the stimulus behind the case for universal screening lies in early intervention. Numerous studies (e.g., Refs. 13, 14, 15, 16, 17) have related improved clinical outcomes to early intervention, providing motivation for diagnosing individuals as early as accurate diagnosis is possible.

The “spectrum” nature of ASD coupled with the rapid, highly variable development processes present early in life elevates the challenges of early diagnosis of ASD. Miller et al.^18^ illustrate this point by comparing two hypothetical children:

“An active, verbose child who speaks primarily in stereotyped phrases and is preoccupied with train schedules might be immediately recognized as autistic. Likewise, a child who is nonverbal, does not respond to his name despite normal hearing, and who spins things repetitively might also be immediately recognized as autistic. Both children have underlying impairments in social communication and restricted interests, but the surface presentation is quite different.”

A wealth of psychometric tools available to healthcare professionals aid in diagnosing ASD; however, a biological signature of the disorder promises to lower the age of diagnosis without the challenges associated with a behavioral diagnosis of a developmental disorder. Heterogeneity in typical development patterns limits the earliest age at which ASD is reliably diagnosed; prospective studies on social behaviors such as gaze to faces, shared smiles, and vocalizations to others found that these behaviors were not different at 6 months of age, but group differences began to appear at 12 months.^19^ However, biomarker signatures of ASD, such as imaging of white matter tract organization^20^ and EEG complexity,^21^ have been observed as early as 6 months of age. Recent reports from NeuroPointDX suggest amino acid panels are predictive of ASD status in children aged 4–6 years^22^ and 18–48 months^23^, ^24^ and these promising results suggest extensions down to even younger participants to evaluate the earliest age at which these signatures appear. Such biomarker‐based metrics have been shown to aid in the diagnosis of other disorders traditionally solely diagnosed by behavioral observations such as major depressive disorder.^25^

Biomarkers come with their own set of challenges before they reach clinical translation: less than 0.1% of cancer biomarkers reported in the literature ever enter clinical practice^26^ and scores of genome wide association studies seeking predictive ASD biomarkers have found few significant findings, most of which are specific to individual studies.^27^ Reconciling the “holy grail” potential of successful biomarkers with the poor predictive power among those reported in the literature draws into question the manner in which biomarkers are identified. A better framework is clearly needed for identifying predictive biomarkers that can accurately and differentially diagnose ASD.

Classic biomarker development measures a plethora of candidate biomarkers but evaluates this panel by a series of univariate tests that considers each measurement as independent from all others. Furthermore, the population‐level hypothesis tests that are almost synonymous with this approach are ill‐suited for quantifying the separation of two or more groups.^28^ Multivariate biomarkers evaluated by separating individuals (e.g., C‐statistic, confusion matrix, etc.) have become increasingly popular since they can incorporate many pieces of information to arrive at a diagnosis. However, since they require more parameters than their univariate counterparts, special attention has to be paid to avoid overfitting when investigating multivariate biomarkers.

The folate‐dependent one‐carbon metabolism (FOCM) and transsulfuration (TS) pathways comprise a promising source for a multivariate biomarker for ASD. These pathways incorporate both genetic and environmental factors linked to ASD liability.^29^ The authors have previously developed a multivariate biomarker, based upon Fisher Discriminant Analysis (FDA) for ASD that achieved a positive predictive value and negative predictive value of 97.6 and 96.1%, respectively.^29^ This investigation will be extended in this article in two different directions: (a) By comparing univariate analysis with four different multivariate methods on FOCM/TS data for ASD biomarker development to ensure that the identified results are not restricted to FDA and (b) to test and validate multivariate FOCM/TS biomarkers on data collected from a new cohort of ASD participants.

## 2 MATERIALS AND METHODS

### 2.1 Training data

The training data used in this study have been published previously.^29^ Briefly, data come from the Arkansas Children's Hospital Research Institute's autism IMAGE study and detailed study design, inclusion/exclusion criteria, and demographic information have been published elsewhere.^30^ Children between the ages of 3 and 10 years were recruited locally and enrolled to assess levels of oxidative stress. ASD was assessed by a diagnosis of “Autistic Disorder” as defined in the *Diagnostic and Statistical Manual for Mental Disorders, Fourth Edition*, the Autism Diagnostic Observation Schedule (ADOS), and/or the Childhood Autism Rating Scales (CARS; score > 30); children with other diagnoses on the autism spectrum or rare genetic diseases with similar symptoms to ASD (e.g., fragile X syndrome) were not eligible for participation. TD participants had no medical history of behavioral or neurologic abnormalities by parent report. FOCM/TS metabolites from 83 and 76 case (ASD) and age‐matched typically‐developing (TD) control children, respectively, were used for classification. The protocol was approved by the Institutional Review Board (IRB) at the University of Arkansas for Medical Sciences and all parents signed informed consent.

### 2.2 Validation data

The validation data are taken at baseline from three previously published studies investigating pharmaceutical interventions to normalize metabolic abnormalities of children with ASD^31^: (1) a combination of methylcobalamin and low dose folinic acid^32^, ^33^ (2) high dose folinic acid,^34^ and (3) sapropternin.^35^ Given that these studies all focused on evaluating treatment strategies for ASD, all participants had a confirmed diagnosis of ASD. FOCM/TS metabolites were available for 154 (76% male) participants with ASD with a mean age of 8.8 years (range 2–17 years). These ages are different than reported by Delhey et al.^34^ because this study only required that measurements be available at baseline, rather than both at baseline as well as the conclusion of the treatment phase. Furthermore, stratifying patients by age or gender did not reveal any differences in the univariate metabolite distributions. The first two studies were approved by the IRB at the University of Arkansas for Medical Sciences and the third study was approved by the IRB at the University of Texas Health Science Center at Houston. All parents gave written, signed consent and patients provided assent when appropriate.

### 2.3 Metabolites

The metabolites under investigation are presented in Table 1 and additional details of these measurements and derivations are presented in Melnyk, et al.^30^ This is only a subset of the measurements investigated previously^29^ because “% DNA methylation” and “8‐OHG” were absent from the validation data set and were therefore removed from this study to ensure that a consistent set of metabolites are used for training and testing.

**Table 1:** Variable identifiers (ID) and names

ID	Variable name	ID	Variable name
$x_{1}$	Methionine	$x_{12}$	GSSG
$x_{2}$	SAM	$x_{13}$	fGSH/GSSG
$x_{3}$	SAH	$x_{14}$	tGSH/GSSG
$x_{4}$	SAM/SAH	$x_{15}$	3‐ClT
$x_{5}$	Adenosine	$x_{16}$	3‐NT
$x_{6}$	Homocysteine	$x_{17}$	Tyrosine
$x_{7}$	tCysteine	$x_{18}$	Tryptophane
$x_{8}$	Glu‐Cys	$x_{19}$	fCystine
$x_{9}$	Cys‐Gly	$x_{20}$	fCysteine
$x_{10}$	tGSH	$x_{21}$	fCystine/fCysteine
$x_{11}$	fGSH	$x_{22}$	% oxidized glutathione

### 2.4 Kernel density estimation

Kernel density estimation (KDE) is a nonparametric density estimation technique that overcomes many shortcomings of the common histogram, including discontinuities at bin boundaries, sensitivity with respect to the origin, and zero‐valued outside of a certain range.^36^ In this work, all KDE procedures use Gaussian kernels. The probability density function (PDF) estimates provided by KDE are then used to evaluate both the C‐statistic and misclassification errors at specific one‐sided thresholds on the p value for membership in the ASD class to characterize the various statistical models described below.

### 2.5 Statistical techniques

Multivariate classification for ASD diagnostic status was explored through classification and regression trees, principal component analysis, fisher discriminant analysis, and logistic regression. The presented techniques can be extended to classification tasks with more than two classes; however, only binary classification (i.e., classification into two different groups) will be discussed below. Sample x can belong to one of two classes $\Pi_{1}$ and $\Pi_{2}$.

#### 2.5.1 Univariate classification

Perhaps the simplest way to develop a classifier for a diagnostic biomarker is to place a simple threshold on a single measurement. For multivariate data, the modeler would then evaluate each measurement independently and choose the measurement with the best discriminating power. In this work, single measurements are mean‐centered and normalized to unit variance before estimating the PDFs of the ASD and TD groups.

#### 2.5.2 Classification and regression trees

When univariate techniques fall short, the modeler must turn to multivariate techniques (i.e., techniques that incorporate multiple features to determine the classification). One intuitive extension from the simple univariate classification scheme is to sequentially place thresholds on many variables in the data set. The most common application of this principle is through recursive partitioning via the classification and regression tree (CART) methodology.^37^ Since sequential thresholds are placed on variables and multiple thresholds on the same variable are permitted, CART‐based classifiers are generally nonlinear.

The tree‐growing process begins with a node τ and a node impurity function i(τ). A proposed split s generates two daughter nodes $\tau_{L}$ and $\tau_{R}$ that contain $p_{L}$ and $p_{R}$ proportions of the samples in τ. Defining the node impurity function i(τ) to be the conditional probability that a sample is in $\Pi_{1\;}$, the change in impurity is given by

(1) $$\Delta i\left(s,\tau \right)=i\left(\tau \right)-p_{L}i\left(\tau_{L}\right)-p_{R}i(\tau_{R})$$

and the split with the greatest reduction in impurity over all variables and all thresholds is chosen. This procedure is repeated for each node until each node contains fewer than some minimum splitting threshold. Each terminal node (i.e., a node with no daughter nodes) is associated with either the ASD or TD class. Next, the tree is pruned upwards by estimating the misclassification rate or risk R(τ) of the entire tree versus subtrees with one terminal node removed, regularized by the number of terminal nodes $T_{\tau }$ via parameter α:

(2) $$R_{\alpha }\left(\tau \right)=R\left(\tau \right)+\alpha |T_{\tau }|$$

With α chosen via 10‐fold cross‐validation (see section “Avoiding Overfitting: Cross‐validation”), the tree that minimizes $R_{\alpha }\left(\tau \right)$ is chosen as the final tree. In other words, the tree that balances the misclassification rate with the number of terminal nodes/splits is chosen as the final tree. The classification trees were carried out using the R package “rpart”^38^ with $i\left(\tau \right)$ defined by the Gini index and the minimum splitting threshold set to eight.

#### 2.5.3 Principal component analysis

Rather than making many sequential decisions through CART methodology, the original data can be projected onto a line and a single binary threshold can be applied to the resulting univariate score. Under the naïve assumption that the most favorable projection for separating the two classes coincides with the projection with maximum variance, principal component analysis (PCA) can be used to establish the projection direction. Principal components are orthogonal and ordered such that the first principal component explains the majority of the total variance.^39^ In this work, PCA is applied to the panel of metabolites with unknown class membership. Then, the density of the first principal component for each class is estimated via KDE to construct a binary classifier. A threshold is applied to the first principal component at an accepted misclassification rate of the ASD class to develop a simple binary classifier. Although multiple principal components could be used in a classifier, this work focuses on the first principal component to better compare with the other projection‐based methods discussed below and to avoid overfitting by introducing more variables. The PCA analysis was conducted in MATLAB.

#### 2.5.4 Fisher discriminant analysis

Since the data contains class labels (i.e., ASD or TD) for each panel of measurements, a potentially better way to determine the projection direction would directly use the class membership information to maximally separate the distance between the two classes of data. FDA^40^ achieves this by maximizing the ratio of the between‐class scatter to the within‐class scatter of the two classes. In other words, the mean of the ASD group and that of the TD group are separated as far as possible, while simultaneously minimizing the variance within each group. As in the PCA analysis, the densities of the FDA scores of the two classes are estimated via KDE and a threshold is applied at an accepted misclassification rate of the ASD class to develop a simple binary classifier. The FDA analysis was conducted through routines developed in‐house in MATLAB.

#### 2.5.5 Logistic regression

Using a probabilistic approach, the conditional probability of membership in class i given the data point is given as $p\left(\Pi_{i}|x\right)$. The odds ratio that $\Pi_{1}$ is the correct class is then

(3) $$\frac{p\left(\Pi_{1}|x\right)}{p\left(\Pi_{2}|x\right)}=\frac{p\left(\Pi_{1}|x\right)}{1-p\left(\Pi_{1}|x\right)}$$

Logistic regression (LR) then assumes that the logarithm of this odds ratio can be modeled as a linear function of x

(4) $$L\left(x\right)=\log\left(\frac{p\left(\Pi_{1}|x\right)}{1-p\left(\Pi_{1}|x\right)}\right)={w_{0}+w}^{T}x$$

where w and $w_{0}$ are estimated through maximum likelihood estimation (MLE). Then, the probability distributions can be directly determined as $p\left(\Pi_{1}|x\right)=e^{L\left(x\right)}/(1-e^{L\left(x\right)})$ without the need for KDE.

There are many theoretical and experimental studies comparing FDA and LR, resulting in the following outcomes: (a) LR performs better than FDA for non‐normal data,^41^ (b) LR requires more data to achieve the same asymptotic error rate as achieved by FDA,^42^ though it is possible for LR to achieve its asymptotic error rate with less training data than FDA,^43^ and (c) MLE of parameters in LR is unstable for separable data, requiring regularization approaches or alternatives to MLE. In practice, these algorithms can be compared on specific data sets to determine the best algorithm for each scenario. LR analysis was conducted using the “glm” function in R.

### 2.6 Avoiding overfitting

Multivariate techniques promise more accurate biomarkers as they incorporate many measurements into a diagnosis; however, these techniques can also suffer from overfitting where the model fits the training data exceptionally well but extends poorly to new data sets. Overfitting can occur when some of the included variables are uninformative or correlated with other variables. Therefore, variable selection is employed to choose a minimal set of measurements for use in the classification procedure. In this work, all combinations of variables were evaluated and the combination with the highest C‐statistic was chosen for a fixed number of variables. While this presents a large number of variable combinations, this task was completed on a standard laptop computer and runtime was no more than an hour for a fixed number of variables. As the number of variables and/or complexity of the classifier increases, filter methods, such as the report by Li et al.^44^ that used this training data, can be used to separate the variable selection and classification problems for improved computational efficiency. Furthermore, validation strategies such as testing on separate validation data sets and cross‐validation are needed to mitigate overfitting and estimate the model's predictive power.

#### 2.6.1 Validation set

A validation set directly estimates the model's predictive power by evaluating the model on data which it has not yet seen (i.e., was not used for training). A validation set can either be collected after the initial model was trained, or more commonly, a single data set is split into training and validation sets at the start of the modeling procedure. Here, a new data set was incorporated to validate previously developed algorithms.

#### 2.6.2 Cross‐validation

Since initial clinical investigations aiming to uncover diagnostic biomarkers usually obtain measurements on a small number of individuals (e.g., less than 100 participants per group), setting aside too large a portion of data for validation does not leave sufficient samples for proper model identification. Therefore, the estimated predictive power is artificially low. To retain as many samples as possible in the training set, k‐fold cross‐validation can be used where the samples are divided into k groups and one group is left out at a time, leaving the remaining k−1 groups available for model training. Then the model is validated on the group that is left out and the procedure is repeated such that every group is successively left out, so every sample is validated in a statistically independent manner. Here, a variation of k‐fold cross‐validation known as leave‐one‐out cross‐validation is used such that k is equal to the number of samples, and this cross‐validation strategy is used to estimate the model's predictive power within the training data set.

#### 2.6.3 Binary classification for projection‐based methods

The PCA, FDA, and LR models all define a projection direction, but classification requires transforming the continuous‐valued scores into a single ASD or TD classification. Throughout this work the threshold β is chosen to fix the estimated probability that a TD participant will be misclassified as ASD.

## 3 RESULTS

The different classification methods are first compared with regard to their performance on the training data. Variable selection is used in some cases to determine final FDA and LR models. Once these final models are identified, the models with the highest classification accuracy are evaluated on the validation data.

### 3.1 Univariate modeling

**Figure 1:**
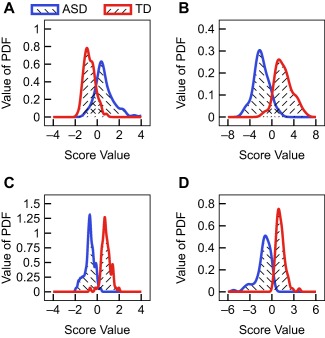
Comparison of fitted PDFs for (a) univariate, (b) PCA‐all, (c) FDA‐all, and (d) LR‐all

Since most biomarker studies focus solely on univariate methods, each variable is first explored individually on the training data for their potential predictive power. Univariate classifiers were evaluated via the C‐statistic and cross‐validated misclassification errors at thresholds on the p value for membership in the ASD class. These results point to “% oxidized glutathione” as the variable with the greatest discriminatory power (Table 1; Figure 1a). At a threshold of β=0.10, this univariate classifier achieves a cross‐validated misclassification of 6/76 and 18/83 for the TD and ASD participants, respectively. While not sufficient for a diagnostic biomarker, this univariate classifier was used as a baseline for comparison in the multivariate analyses.

### 3.2 Performance of projection methods on the training data

Next, multiple measurements were combined in linear, projection‐based classifiers. PCA was employed first as it does not make use of class labels in determining the projection‐direction. Using all variables, PCA obtained a C‐statistic of 0.9706 on the training data. Furthermore, a binary threshold at β≥0.10 membership in the TD class to be classified as TD results in misclassifying only 6/76 and 3/83 of the TD and ASD participants, respectively (Table 1; Figure 1b). This result indicates that the direction of highest variance in the training data also separates the data fairly well for this problem.

Using the group membership in developing a linear, multivariate classifier through FDA or LR promises to further enhance the separation and these methods provide a more solid statistical background for choosing the projection direction than using the direction obtained from PCA for classification. Using all variables, both the FDA and LR models achieve a fitted C‐statistic of > 0.99 (Table 1; Figure 1c,d). These results suggest that including the group membership in determining the separation direction improves the classification performance, as expected, which is also reflected by the low misclassification numbers of 7/159 and 13/159 at a threshold of β=0.05 for the FDA‐all and LR‐all models, respectively.

### 3.3 Classification trees

**Figure 2:**
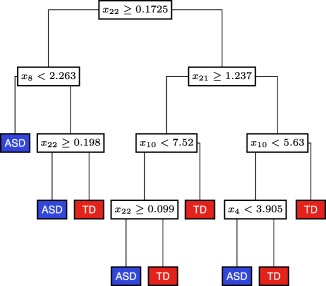
Final CART tree fitted to the training data

Instead of investigating a single binary threshold on single variables or scores, multiple thresholds on multiple variables were investigated using the CART methodology. This simple nonlinear classifier was used to investigate the advantages of moving from linear to nonlinear descriptions of the data. After growing and pruning a classification tree, the resulting model fitted to the training data included five variables ( $x_{22}=$ % oxidized, $x_{8}=$ Glu‐Cys, $x_{21}=$ fCystine/fCysteine, $x_{10}=$ tGSH, and $x_{4}=$ SAM/SAH) and this final tree is illustrated in Figure 2. This model achieves a fitted misclassification of 3/76 and 3/83 for TD and ASD participants, respectively. Since the CART methodology can change both the model structure and parameters upon cross‐validation, only fitted results are provided for the classification tree.

### 3.4 Variable selection for the FDA and LR models

Since previous analysis and the CART results suggest that only a subset of variables is needed to effectively classify the participants into ASD and TD cohorts, variable selection was performed for both the FDA and LR models. All variables combinations were evaluated for the fitted C‐statistic. The C‐statistic began to saturate at five variables for the FDA model and at six variables for the LR model. The cross‐validated performance of these models with only a subset of the variables (FDA‐sub and LR‐sub) is provided in Table 2. The fitted C‐statistic and cross‐validation results are similar for FDA‐sub and LR‐sub and the variables selected between these two models overlap substantially. These diagnostics indicate that these models are able to find similar patterns in the data that include the most important variables for classification. At a threshold of β=0.05, using five variables only slightly increased the cross‐validated misclassification error from 7/159 in the FDA‐all model to 8/159 in the FDA‐sub model, indicating that many of the variables provide limited or redundant information for classification of the ASD and TD cohorts. For the LR models at a threshold of β=0.05, decreasing the number of variables decreased the cross‐validated misclassification error from 13/159 to 10/159, providing further evidence that including too many variables can lead to overfitting of these models to the training data.

**Table 2:** Cross‐validation comparison of univariate and projection‐based multivariate classifiers

		Fitted	Cross‐validated confusion matrix				
Classifier	Variables	C‐statistic	*β*	TP	FP	FN	TN
Univariate	$x_{22}$	0.9159	0.01	23	1	60	75
			0.05	47	3	36	73
			0.10	65	6	18	70
			0.20	71	13	12	63
PCA‐all	All	0.9706	0.01	56	1	27	75
			0.05	77	3	6	73
			0.10	80	6	3	70
			0.20	80	16	3	60
FDA‐all	All	0.9915	0.01	50	1	33	75
			0.05	81	5	2	71
			0.10	81	13	2	63
			0.20	82	19	1	57
FDA‐sub	$x_{4},\;x_{8},\;x_{12},\;x_{21},\;x_{22}$	0.9711	0.01	38	1	45	75
			0.05	78	3	5	73
			0.10	81	8	2	68
			0.20	81	16	2	60
LR‐all	All	0.9972	0.01	73	6	10	70
			0.05	78	8	5	68
			0.10	79	11	4	65
			0.20	81	19	2	57
LR‐sub	$x_{4},\;x_{8},\;x_{10},\;x_{17},\;x_{21},\;x_{22}$	0.9757	0.01	21	1	62	75
			0.05	79	6	4	70
			0.10	81	8	2	68
			0.20	81	15	2	61

The threshold β is the percent membership in the TD class. TP refers to True Positive, FP to False Positive, FN to False Negative, and TN to True Negative.

### 3.5 Validation performance

**Figure 3:**
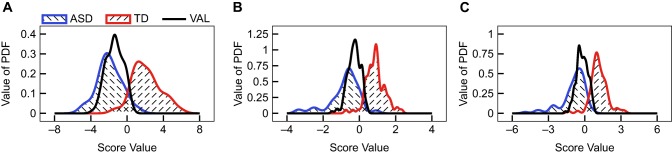
Predictions of the ASD validation data from the (a) PCA‐all, (b) FDA‐sub, and (c) LR‐sub models. Note that the validation data, “VAL,” only consists of data from a set of children with an ASD diagnosis and therefore significant overlap between VAL and ASD is expected and desired

All previous sections only made use of the training data. Here, the final classifiers were then fitted to the training data set and evaluated on the validation set. It should be noted that the validation set only includes participants with an ASD diagnosis and so the validation can only test for true positive and false negative ASD classifications. Therefore, all classifiers are evaluated at a threshold of β=0.05 percent membership in the TD class to provide some measure of the expected false positive rate for a given classifier. The PDFs of the predicted ASD class in the validation set are provided in Figure 3. With a threshold of β=0.05 membership in the TD class to be classified as TD, the predicted binary classification is provided in Table 3. The univariate model produces a high false negative rate of 86/154 at β=0.05 which can be reduced to 42/154 at β=0.10. These results provide further evidence that univariate models are insufficient for classifying ASD and TD participants. In contrast, the FDA‐sub model produces a false negative rate of only 19/154 at β=0.05, highlighting the advantages of using multiple FOCM/TS variables in classifying participants as ASD versus TD. All of the linear projection‐based methods perform reasonably well on the validation set, but the FDA‐sub model has the lowest validation error. Furthermore, the linear methods are sufficient for separating these two cohorts, supported by the 38/154 false negative rate produced by the CART model.

**Table 3:** Validation performance for the final biomarker models

Classifier	Variables	*β*	TP	FN
Univariate	$x_{22}$	0.05	68	86
		0.10	112	42
PCA‐all	All	0.05	128	26
		0.10	149	5
FDA‐sub	$x_{4},\;x_{8},\;x_{12},\;x_{21},\;x_{22}$	0.05	135	19
		0.10	150	4
LR‐sub	$x_{4},\;x_{8},\;x_{10},\;x_{17},\;x_{21},\;x_{22}$	0.05	122	32
		0.10	139	15
CART	$x_{4},\;x_{8},\;x_{10},\;x_{21},\;x_{22}$	–	116	38

TP refers to True Positive and FN to False Negative. False Positive and True Negative are not needed for the validation set as it only consists of data from a set of children with an ASD diagnosis.

## 4 DISCUSSION

This study compares univariate analysis to four different multivariate statistical techniques through a cross‐validation study for the classification of ASD versus TD cohorts. The final models are then fitted to the training data and the resulting models are tested on new validation data, compiled from three previous studies. Since many biomarker studies evaluate each variable individually, the best univariate model was used for comparison. The univariate model using % oxidized indicated a modest separation between the ASD and TD cohorts with a very high false positive rate of 86/154 at an expected false negative rate of 5%. This false positive number reduces to 42/154 if the expected false negative rate is raised to 10%; however, this classification accuracy is not sufficient for developing a diagnostic test.

As an alternative to univariate classifiers, multivariate techniques were introduced to demonstrate the improved performance obtained from simultaneously using data from multiple variables. All multivariate classification techniques performed similarly well on the training data set and they far outperformed the univariate classifier by a wide margin for all four values of the expected false negative rate that were investigated ( β= 1%, 5%, 10%, 20%). When these classifiers were applied to the validation set then the general trend was upheld, but some differences between the methods emerged: The first principal component of the data was able to sufficiently separate the data (PCA‐all produced a false positive rate of 26/154 at an expected false negative rate of 5%), indicating that most of the variation present in the data can be attributed to the differences in the ASD and TD cohorts. The FDA‐sub model had the best validation performance with a false positive rate of 19/154 at an expected false negative rate of 5%. As expected, these misclassification rates are higher than the false negative rate of 3.6% at a false positive rate of 2.6% found previously^29^ due in part to the absence of “% DNA methylation” and “8‐OHG” in the validation data, two of the most important variables for separating ASD and TD cohorts.^29^, ^44^ CART and LR of a subset of metabolites produced results that were slightly worse than PCA with false positive rates of 38/154 and 32/154, respectively; both of these results are at an expected false negative rate of 5%. These results also indicate that linear combinations of variables are sufficient for separation of ASD and TD cohorts using these FOCM/TS metabolites and nonlinear techniques are likely not warranted for these data sets.

When the expected false negative rate was increased to a level of 10% then the true positive predictions of FDA‐sub, PCA‐all, and LR‐sub improved as expected. CART does not allow for a single parameter to tune the classification in a way analogous to the projection‐based methods, so the number of true positive rates for CART remains the same. Among these methods FDA‐sub performs slightly better than the other techniques on the validation set, reaching true positive predictions as high as 150/154. That being said, a false negative rate of 10% is quite large for a diagnostic test and as such the results for β=0.05 as discussed in the previous paragraph are more relevant than the results for β=0.1.

The large number of classification algorithms found in the literature prohibits an exhaustive comparison of all the available techniques. In particular, classification trees are not the only method for nonlinear classification, but they are generally easier to convey to the general scientific audience than the popular kernel methods that can extend PCA, FDA, and LR to nonlinear classification or various neural network strategies. Nonlinear classification techniques are usually applied in domains where data are easier to collect, and data sets are generally larger; therefore, these methods are likely not ideal for early‐stage clinical data. Additionally, at least for the data sets under study in this work, we did not find advantages of using nonlinear classification techniques.

Aside from the comparison of the algorithms it is equally important to discuss the finding of which metabolites where identified as contributing the most to the predictive performance. Univariate analysis identified $x_{22}$, “% oxidized glutathione,” as the most important variable. This variable also appears in the FDA‐sub, LR‐sub, and CART models further highlighting the importance of the contribution of “% oxidized glutathione” even when multivariate analysis is used. In addition to this variable the subsets chosen for FDA‐sub, LR‐sub, and CART also have three other variables in common: $x_{4}$: “SAM/SAH,” $x_{8}$: “Glu‐Cys,” and $x_{21}$: “fCystine/fCysteine.” Furthermore, “% oxidized glutathione” highlights the importance of oxidative stress for classification while the “SAM/SAH” ratio is directly linked to DNA methylation and epigenetic components. As suggested previously,^29^, ^44^ it is important to include variables that account for both FOCM (DNA methylation) and TS (oxidative stress) pathways in separating ASD from TD cohorts and this is what the performed analysis returned regardless of which technique was used.

Although the results of this study are promising, there are several limitations that should be considered in future studies.

1. Including the same variables in the training and validation data. Previous analyses found that “% DNA methylation” and “8‐OHG” were two of the most important variables for separation,^29^, ^44^ but these data were not present in the validation set. Future studies should include these variables to allow for the highest possible classification accuracy as these classifiers are considered for clinical translation into a diagnostic test.
2. Including both ASD and TD populations. The validation set included in this study provides a first attempt to validate previous findings with a new data set of similar size, but it only includes ASD participants. While it would be preferable to have a validation set that includes measurements from ASD and TD cohorts, as compared to only from an ASD cohort, these data do not currently exist aside from the one which was used for training here. Future studies should collect additional data and evaluate both ASD and TD populations to confirm separation of these two groups. Finally, the slight improvements in classification obtained by the FDA‐sub classifier in comparison with the other methods tested herein should be reaffirmed after evaluating these classifiers on additional TD data.
3. Analyzing younger participants. The training and validation set comprise cohorts of 3–10 years and 2–17 years, respectively. However, a young cohort comprised mainly of participants younger than about 3 years would provide more compelling evidence toward using classifiers such as these to aid in the diagnosis of ASD.

Successfully addressing these limitations would help to solidify the ability of multivariate statistical tools based on FOCM/TS measurements to accurately separate ASD and TD participants and ultimately allow these classifiers to be translated into the clinic.

## 5 CONCLUSIONS

This study compared several different classification techniques applied to FOCM/TS metabolites with the purpose of evaluating these metabolites and the analysis techniques as potential biomarkers for ASD. PCA, FDA, LR, and CART models were evaluated in a cross‐validation approach on a training data set and then used to predict validation data comprised of 154 new ASD participants and all of these classifiers achieved satisfactory results. An FDA model using five variables was shown to slightly outperform the other models on this new validation data set. While these results look very promising and serve as a partial validation of our previous investigation, future studies should investigate larger cohorts of ASD and TD populations across multiple clinical sites to further support the indication for impaired DNA methylation and increased oxidative stress associated with ASD.

## Supporting information

Additional Supporting Information may be found online in the supporting information tab for this article.

Supporting Information 1
